# Exploration of Risk Factors for Type 2 Diabetes among Arabs in Israel

**DOI:** 10.5334/aogh.2350

**Published:** 2019-05-10

**Authors:** Rajech Sharkia, Ahmad Sheikh-Muhammad, Muhammad Mahajnah, Mohammad Khatib, Abdelnaser Zalan

**Affiliations:** 1 The Triangle Regional Research and Development Center, Kfar-Qari’-30075, IL; 2 Beit-Berl Academic College, Beit-Berl 44905, IL; 3 The Galilee Society – The Arabs National Society for Research and Health Services, Shefa-Amr, IL; 4 Child Neurology and Development Center, Hillel-Yaffe Medical Center, Hadera, IL; 5 Ruth and Bruce Rappaport Faculty of Medicine, Haifa, IL

## Abstract

**Background:**

Type 2 Diabetes Mellitus (T2DM) is becoming increasingly prevalent and is considered to be a major public health threat worldwide. Behavioral and sociodemographic factors associated with T2DM vary within different societies.

**Objective:**

The aim of this study is to determine the various behavioral and sociodemographic factors associated with T2DM in the Arab society in Israel.

**Methods:**

A cross-sectional study was conducted based on data from 1,894 residents over the age of 21 belonging to the Arab population in Israel. The data collected from the subjects were subjected to statistical analyses using the SPSS program.

**Findings:**

Of the total sample population, 13.7% were found to be affected with T2DM. The prevalence of T2DM increased sharply in the successive age groups of both men and women. The prevalence of T2DM was found to increase progressively particularly in women with an increase in BMI (~20%, 37% and 44% respectively), while, in men it increased sharply (from 25% to ~50%) until a BMI of 29.9; it then decreased drastically (to ~24%) for a BMI of ≥30. About 85% of the men affected with T2DM were physically inactive, while 97% of the affected women were physically inactive. Almost half of the participants with diabetes have a family history of the disease in both genders. In the multivariate analysis, it was found that age, obesity, physical inactivity and family history of the disease were the significant factors associated with the prevalence of diabetes.

**Conclusions:**

It could be concluded that age, obesity, family history and physical inactivity were the significant factors associated with the prevalence of T2DM within the Arab society in Israel.

## Introduction

Type 2 Diabetes Mellitus (T2DM) is a major public health threat having an increasing prevalence among the general population worldwide [1]. This disease is increasing rapidly in developing as well as in developed countries; furthermore, it is even found to be increasing to a higher extent in the Arab world [2]. Some Arab countries have the highest prevalence of diabetes (in adults aged 20 to 79), ranging from 19% to 21% [3]. Not only does this chronic disease affect the patients themselves, it also causes considerable emotional and financial suffering among the families, relatives and all of society [4].

T2DM is a polygenic disorder involving interactions between genetic and environmental risk factors, such as air pollution, traffic-related pollutants [5], organic land pollutants such as pesticides and herbicides [6], and residential noise [7,8], which result in the underlying pathophysiology of hepatic and muscle insulin resistance, and subsequent beta-cell failure [9,10,11]. Several studies suggest a link between environmental, behavioral and health outcomes that are closely related to T2DM, such as obesity, cardiovascular diseases, hypertension, metabolic syndrome and physical activity [12,13]. It was shown that individual-level socioeconomic, demographic and behavioral factors are important predictors of T2DM [14].

Regarding Arab societies, behavioral and sociodemographic factors play a vital role in the increasing rate of T2DM [2]. It was found that obesity, rapid urbanization and lack of exercise are key determinants of the large increase in the rate of T2DM in the Arab world [15]. This is believed to be a logical outcome of the rapid economic growth, which carried with it the burden of greater reliance on mechanization, a proliferation of western-style fast food and access to cheap migrant labor, thus resulting in greater opportunities for sedentary lifestyles, especially among the younger population. These environmental factors are fueling the emerging epidemic of type 2 diabetes in Arab countries [2,15].

The Arab population in Israel, which today totals about 1.8 million (according to data from the Central Bureau of Statistics, 2016), is an ethnic group having unique cultural, religious and social characteristics that differ from those of the general population in Israel. This community is characterized by a high rate of consanguineous marriages with a common founder effect [16,17]. Recently, a westernized lifestyle was adopted by most of the Arab population in Israel [18]. It was found that the main causes of death that might contribute to the lower age of Arabs compared to Jews could be due to chronic diseases, especially ischemic heart disease and diabetes [19]. A local study showed that the prevalence of diabetes was 21% in Arabs while it was 12% in Jews, however, Arabs developed diabetes 11 years earlier than Jews [20]. Furthermore, recent studies confirmed the difference in the prevalence of diabetes between Arabs and Jews [21,22]. Our previous study found that the incidence of T2DM increased significantly from 11.3% to 17.7% in the years 2005 and 2015, respectively, with a progressive increase with age in both genders [23]. Since behavioral and sociodemographic factors vary between different societies, it became of vital importance to determine the most relevant factors that apply to our society in these terms. To the best of our knowledge, this issue has not been investigated previously in a comprehensive manner in the Arab community in Israel. Thus, the aim of our current study is to determine the different factors associated with this alarming disease, T2DM, within our community.

## Methods

1. **Study population:** A cross-sectional study was conducted based on data from a total of 1,894 residents out of 2,250 people over the age of 21 years and belonging to the Arab population in Israel who had been contacted for the study (a response rate of 84.2%). A sampling of this nationwide study was carried out using the cluster sampling method. The sample was designed in three stages: selection of enumeration areas in one stratification level; selection of 30 responsive households in the chosen enumeration area; and selection of two persons, a male and a female, aged ≥21 years. This selection was made from each household in the second stage using the spreadsheet (Kish) for random selection. The study population was divided into homogeneous strata, taking into account gender and age group variables. The sample obtained was divided according to gender as men and women; furthermore, they were subdivided into two categories, namely, people with and without diabetes. The various behavioral and sociodemographic factors of the study population were explored in this study. All the participants recruited for the study gave their informed written consent after being provided with an explanation of purpose, conducted in accordance with the declaration of Helsinki.
2. **Data collection:** The data were collected from all the subjects through self-reporting and personal face-to-face interviews using a survey questionnaire prepared specifically for this purpose. The age parameter was divided into three subgroups (21–49 years, 50–64 years and ≥65 years). Height and weight were obtained from the participants directly. Body mass index (BMI in kg/m^2^ units) was also divided into three categories: <25 (normal weight); 25–29.9 (overweight); and ≥30 (obesity). The family’s monthly income level was calculated by dividing the total household income into the number of persons per household. This parameter, in turn, was divided into three groups and expressed in New Israeli Shekels (NIS) *viz*, <2,611 (low), 2,612–3,999 (medium) and ≥4,000 (high). The education parameter was divided into two groups, namely, ≥12 years of education and ≤12 years of education. The locality of residence parameter was divided into two groups: localities with 15,000 inhabitants and above were considered towns and cities; those with less than 15,000 inhabitants were considered villages. The study also included other parameters such as family history of diabetes, physical activity (defined as whether the subject does any type of regular sport or not), smoking and alcohol consumption.
  Information about diabetes was obtained from the participants through interviews by asking specific questions, such as: have you ever been diagnosed by a physician as being diabetic? Participants who answered yes were asked about the type of diabetes and how old he/she was at the time of the diagnosis. The sample excluded subjects who had type 1 diabetes. Questionnaires were completed through face-to-face interviews. The questionnaire had been used in previous studies (socioeconomic survey) in which validity tests showed good results. The fieldwork team was recruited from a group of experienced surveyors. A training course was conducted for these surveyors by the supervisors and the project administrators.
3. **Data and statistical analysis:** The data collected via the questionnaire were used to construct a database with Microsoft Access software. Furthermore, the data were subjected to random spot-checking and verification; they were then imported into the SPSS program for data management and statistical analysis. Frequency tallies were performed on all categorical variables, prevalence rates were determined, and χ^2^ tests were carried out as required. The Kruskal-Wallis test was used to evaluate the significance of the differences observed across age or BMI. Univariate and multivariable logistic regression analyses were performed to examine the factors associated with T2DM. Variables that were associated significantly with T2DM in the univariate analysis were entered into the multivariate analysis. Crude odds ratios (ORs) and 95% CIs were calculated; adjusted odds ratios and 95% CIs were derived from the logistic regression model. Statistical significance was determined at *P* < 0.05.

## Results

Our study includes a total of 1,894 persons over the age of 21. Their characteristics relating to demographic and behavioral variables are presented in Table 1. It was found that the majority of the participants lie in the age range category of 21–49 years, constituting about 71%, while the older age group of 50–64 years had about 18%, and the least participants were found in the 65 years of age and above category (~11%). These percentages were found to be very similar in both genders. Regarding years of education, it was found that majority of the participants had less than 12 years of education (~80%), while just about 20% had academic qualifications, with similar findings for both men and women. Regarding localities of residence, out of all the participants, it was found that slightly more than half (53%) live in cities and towns, and the rest live in villages. More than half of the participants (~51%) belong to families having an income level lower than the average income of the families in Israel, while the rest are distributed similarly between medium- and high-income levels.

**Table 1:** Characteristics of the study sample by gender.

Factors	Men	Women	Total	*P*

	% (N = 929)	% (N = 965)	% (N = 1894)	

Age (Y):				
21–49	72.6 (674)	68.6 (664)	70.6 (1338)	0.199
50–64	16.9 (157)	19.0 (183)	18.0 (340)	
≥65	10.5 (89)	12.2 (118)	11.4 (216)	
Education:				
>12	20.0 (184)	19.5 (186)	19.7 (370)	0.772
≤12	80.0 (735)	80.5 (769)	80.3 (1504)	
Locality Size:				
>15K	53.3 (495)	53.0 (511)	53.1 (1006)	0.886
≤15K	46.7 (434)	47.0 (454)	46.9 (888)	
Income Level:				
Low	49.4 (397)	52.8 (433)	51.1 (830)	0.369
Medium	25.4 (204)	24.1 (198)	24.8 (402)	
High	25.2 (203)	23.0 (189)	24.1 (392)	
BMI:				
<25	28.8 (257)	42.2 (380)	35.6 (637)	0.001
25–29.9	53.9 (480)	37.6 (338)	45.7 (818)	
≥30	17.3 (154)	20.2 (182)	18.8 (336)	
Physical Activity:				
Yes	29.0 (268)	21.2 (204)	25.0 (472)	<0.001
No	71.0 (655)	78.8 (758)	75.0 (1413)	
Smoking:				
Yes	56.6 (522)	10.3 (99)	33.0 (621)	<0.001
No	43.4 (401)	89.7 (862)	67.0 (1263)		
Alcohol Consumption:				
Yes	13.6 (124)	5.8 (55)	9.6 (179)	<0.001
No	86.4 (785)	94.2 (901)	90.4 (1686)	
Type 2 Diabetes:				
Yes	12.1 (111)	15.3 (147)	13.7 (257)	0.038
No	87.9 (810)	84.7 (811)	86.3 (1621)	
Family History:				
Yes	28 (258)	31 (297)	29.6 (556)	0.41
No	72 (664)	69 (661)	70.4 (1322)	

Abbreviations: N – number; Y – Year; K – Thousands; BMI – Body Mass Index.

Most of the participants were found to be overweight and obese (~64%) having a BMI ≥ 25, with a significant difference between men and women (71% vs. 57%). About 75% of the participants were found to be physically inactive with a slight difference between men and women. It was observed that about one third (33%) of the participants were smokers, with a significant difference between men and women (56% and 10% respectively). Less than 10% of the participants consumed alcohol. It is further noticed that the female alcohol consumers were less than half of the male consumers (~6% and ~14% respectively).

According to prevalence of T2DM in the family, about 30% of the participants had a family history of the disease. Of the total sample, 13.7% were found to be affected with T2DM. This prevalence was slightly higher in women than in men.

Table 2 presents the various demographic and health behavioral factors associated with T2DM. It was found that the prevalence of T2DM increased sharply in the successive age groups for both men (23%, 35% and 42% respectively) and women (14%, 38% and 48% respectively). The age group of 65 years and above had the highest prevalence of diabetes among men and women. According to years of education, the vast majority of women with diabetes (98%) and men (88%) had less than 12 years of education. It is evident that the prevalence of T2DM in men decreased progressively with an increase in income level (50%, 31% and 19% respectively), while it decreased sharply in women from low to medium income levels (70% to ~11% respectively); it increased further to 19% in the high income level.

**Table 2:** Demographic and health behavioral factors associated with men and women with or without diabetes.

Factors	Men		Women	

	Without Diabetes	With Diabetes	Without Diabetes	With Diabetes

	% (N = 810)	% (N = 111)	% (N = 811)	% (N = 147)

Age (Y):				
21–49	79.6 (645)	22.5 (25)	78.9 (640)	14.3 (21)
50–64	14.2 (115)	35.1 (39)	15.5 (126)	38.1 (56)
≥65	6.2 (50)	42.3 (47)	5.5 (45)	47.6 (70)
Education:				
>12	21.4 (171)	11.7 (13)	22.7 (182)	2.0 (3)
≤12	78.6 (629)	88.3 (98)	77.3 (620)	98.0 (144)
Locality Size:				
>15K	54.1 (438)	50.5 (56)	51.9 (421)	58.5 (86)
≤15K	45.9 (372)	49.5 (55)	48.1 (390)	41.5 (61)
Income Level:				
Low	49.5 (349)	49.5 (46)	50.1 (353)	70.0 (77)
Medium	24.5 (173)	31.2 (29)	26.1 (184)	10.9 (12)
High	26.0 (183)	19.4 (18)	23.8 (168)	19.1 (21)
BMI:				
<25	29.3 (228)	25.5 (27)	46.2 (351)	19.5 (26)
25–29.9	54.2 (421)	50.9 (54)	37.9 (288)	36.8 (49)
≥30	16.5 (128)	23.6 (25)	15.9 (121)	43.6 (56)
Physical Activity:				
Yes	31.1 (250)	15.3 (17)	24.7 (200)	2.7 (4)
No	68.9 (554)	84.7 (4)	75.3 (609)	97.3 (142)
Smoking:				
Yes	57.6 (463)	48.6 (54)	10.4 (84)	9.6 (14)
No	42.4 (341)	51.4 (57)	89.6 (724)	90.4 (132)
Alcohol Consumption:				
Yes	15.0 (119)	3.7 (4)	6.7 (54)	0.7 (1)
No	85.0 (675)	96.3 (105)	93.3 (749)	99.3 (145)
Family History:				
Yes	25.9 (210)	46.2 (51)	27.6 (224)	49.7 (73)
No	74.1 (600)	53.8 (60)	72.4 (587)	50.3 (74)

Abbreviations: N – number; Y – Year; K – Thousands; BMI – Body Mass Index.

The prevalence of T2DM was found to increase progressively with an increase in BMI, particularly in women (~20%, 37% and 44% respectively), while in men it increased sharply (from 25% to ~50%) until a BMI of 29.9; it then decreased drastically (to ~24%) for a BMI of 30 and above. It is clear that obese women tend to have an almost two-fold higher prevalence of T2DM than their male counterparts (44% vs. 24%). Regarding the physical activity factor, it was found that about 85% of the men affected with T2DM were physically inactive, while 97% of the affected women were physically inactive. On the other hand, this factor was found to differ significantly between the participants with and without diabetes in both genders. About half of the cases with diabetes had a family history of the disease in both genders. The results indicated that locality of residence, smoking and alcohol consumption were not associated with the prevalence of T2DM in both genders.

Through a multivariate analysis, as shown in Table 3, it was found that age, obesity (BMI > 30), physical inactivity and family history of the disease were the significant factors associated with the prevalence of diabetes.

**Table 3:** Results of the multivariate analysis for diabetes.

Factors	Crude		Adjusted	

	OR (95% CI)	*P*	OR (95% CI)	*P*

Age group (Y):		<0.001		<0.001
21–49	1		1	
50–64	11.01 (7.54–16.07)	<0.001	8.41 (5.37–13.19)	<0.001
≥65	34.40 (23.06–51.31)	<0.001	32.58 (19.71–53.83)	<0.001
BMI:		<0.001		0.006
<25	1		1	
25–29.9	1.58 (1.12–2.25)	0.009	1.15 (0.73–1.82)	0.553
≥30	3.64 (2.50–5.30)	<0.001	2.11 (1.27–3.49)	0.004
Physical Activity:		<0.001		0.046
Yes	1		1	
No	4.35 (2.75–6.88)	<0.001	1.82 (1.01–3.29)	0.046
Smoking:		0.018		0.075
No	1		1	
Yes	0.70 (0.52–0.94)	0.018	1.44 (0.94–2.17)	0.075
Family history:		<0.001		0.023
No	1		1	
Yes	4.33 (2.78–7.21)	<0.001	1.60 (1.04 – 2.46)	0.023
Income:		0.016		0.920
(high)	1		1	
(medium)	1.03 (0.65–1.64)	0.889	0.89 (0.50–1.58)	0.697
(low)	1.58 (1.08–2.31)	0.020	0.97 (0.60–1.17)	0.892
Alcohol Consumption:		<0.001		0.064
No	1		1	
Yes	0.16 (0.07–0.40)	<0.001	0.40 (0.15–1.06)	0.064
Education:		<0.001		0.079
>12	1		1	
≤12	4.27 (2.54–7.19)	<0.001	1.82 (0.93–3.58)	0.079
Locality Size:		0.540		0.302
>15K	1		1	
≤15K	0.92 (0.71–1.20)	0.541	1.22 (0.84–1.77)	0.302

Abbreviations: N – number; Y – Year; K – Thousands; BMI – Body Mass Index.

## Discussion

Diabetes has been recognized as being one of the major health burdens worldwide, therefore, it poses a considerable challenge to the human race. The results obtained by our study demonstrated that age of the participants was one of the risk factors associated with diabetes, increasing continuously in the successive age groups in both men and women. The ≥65 age group had the highest prevalence of diabetes in both genders; these findings were in concordance with other studies [24]. It was further found that in a local study, Arabs in Israel were affected with T2DM at a younger age than Jews and 25% of the Arab population was diagnosed with T2DM by the age of 57 compared to the age of 68 in the Jewish population [20]. Additionally, this pattern was also noticed in various Arab societies, e.g. in Kuwait [25].

The current results also reflected that a higher educational level is accompanied by a lower prevalence of T2DM. This is in agreement with a study that found that educational attainment is systematically and positively related to time of diabetes onset. An explanation for more highly educated persons being healthier can be attributed to higher income, cognitive ability and other factors, including time and risk preferences, and self-control [26]. Our study also showed that more than half of the participants belonged to families having a lower income than the average income of families in Israel. This finding is similar to that of a study carried out in Canada, suggesting that many cases of diabetes among low- and middle-income residents could be preventable if equitable measures are taken to reduce their financial gap from higher income families [27].

It has been well-documented that health behavioral factors were found to be important indicators of the incidence of T2DM [12,13]. Our study indicated that the prevalence of T2DM was associated with being overweight and obese in both men and women. Therefore, being overweight and obese are key contributors to the global prevalence of diabetes, affecting not only developed countries but also developing countries [28]. T2DM is considered to be an excellent example of comorbidity associated with obesity. However, the molecular mechanism of how being overweight and obese induce the development of other diseases has not been fully understood [29]. Many other studies have documented that the global rise in obesity, sedentary lifestyles and an ageing population have quadrupled the incidence and prevalence of type 2 diabetes [30]. Furthermore, our results clearly indicated that the majority of patients with diabetes were associated with both physically inactive men and women. This demonstrates that physical inactivity increases the incidence of T2DM in both genders with a particularly high impact in women. Randomized clinical trials around the world have shown that physical activity promotion programs combined with diet control could prevent or delay progression to type 2 diabetes among persons at increased risk [31,32]. Therefore, we suggest that adopting an active lifestyle with a careful and well-controlled diet program could restrict the prevalence of T2DM.

The present study elucidated that family history is associated with the prevalence of T2DM. This finding is in agreement with other studies showing a relationship between family history and the incidence of T2DM [20,33]. Since the Arab community is characterized by a high rate of consanguineous marriages with a common founder effect [17], family history is expected to have an effect on the prevalence of T2DM, thus indicating genetic background involvement with the disease. Our cross-sectional study could represent Arab society in Israel, but a limitation could have been induced since our data was self-reported by the participants themselves (height, weight, income etc.), which could have some degree of subjectivity.

## Conclusions

In summary, it was found that age, obesity, family history and physical inactivity were the significant factors associated with the prevalence of T2DM among Arab society in Israel. On the other hand, education and income levels may have an additional effect in increasing the prevalence of T2DM. It could be concluded that T2DM is the outcome of the interaction between sociodemographic and health behavioral factors within our society.
